# Integrating informal and formal care: An innovative, scalable program blueprint

**DOI:** 10.1177/08404704241292329

**Published:** 2024-11-16

**Authors:** Deborah Sattler, Michelle Howard, Doris Nessim, Marg McAlister, Lisa Dolovich

**Affiliations:** 1 426874 Hospital and Palliative Care Ontario, Toronto, Ontario, Canada.; 2 3710 McMaster University, Hamilton, Ontario, Canada.; 3 42301 MMC Consulting, London, Ontario, Canada.; 4 7938 University of Toronto, Toronto, Ontario, Canada.

## Abstract

People have infinite needs, including illness prevention, wellness, self-care, practical support, and quality of life. This article describes community-based, informal care programs that help people identify their needs, set goals, and organize networks of care to address their needs holistically in a way that can also significantly reduce healthcare costs. Approaches can be customized for primary care, home and community, hospice, and other care sectors to facilitate low-cost, high impact adoption. We provide a blueprint for programs that integrate informal and formal care across social, physical, and mental health domains as a key part of healthcare system transformation.

## Background

### System challenges

Healthcare systems worldwide face tremendous pressure to improve both access to care and health outcomes while containing costs. Despite high per capita healthcare spending, Canada trails on many key health outcomes and supply indicators,^1^ with high levels of unmet need also reported.^2^ Research confirms that the gaps in care of most concern to people in the community and their caregivers relate to practical support, self-care, social relationships, and activities to manage issues such as declining health, increasing disability, loneliness, household tasks, coming to terms with end of life, and bereavement.^3,4^

Formal healthcare systems alone are insufficient and ill-equipped and do not have the resources to address patients’ social, preventative, and practical care needs.^5^ This has serious human consequences for individuals, communities, and society. People with poor social health or who lack naturally occurring informal care networks are more likely to have one or more chronic diseases, higher disease burden, live longer in a state of dependency or disability, and die prematurely.^6-8^ Additionally, seniors who live alone, who are lonely or isolated, or whose caregivers feel distressed have higher odds of placement in long-term care.^9^ People who are isolated are more likely to visit their doctor,^10^ have higher use of medication,^11^ higher incidence of falls,^12^ and increased risk factors for long-term care.^9^ Social isolation is associated with increased emergency department encounters, longer hospital stays, adverse outcomes during hospital stays, and an increased likelihood of dying in the hospital.^10,13^ Social isolation thus negatively impacts healthcare sustainability. Economic studies in both the United States^14^ and the United Kingdom^21^ suggest that healthcare systems likely spend thousands of dollars more each year in excess costs for each socially isolated patient compared to the per capita average after adjusting for other factors. As population ageing continues, social challenges will likely intensify, increasing avoidable healthcare system utilization and costs.

These issues affect a large number of Canadians. With changing social structures and demographics, many Canadians live alone, limiting access to social and informal care resources.^15^ One in three or more Canadians across all age and sociodemographic groups and at all stages of the health and disease spectrum are affected by loneliness or isolation.^16^ People who are vulnerable or marginalized have less access to care and experience some of the poorest health outcomes.^17,18^

### The importance of informal care

Care pertains to the practical and emotional support provided to maintain or improve well-being, alleviate symptoms, and reduce frailty or vulnerability, which can come from formal services, informal carers, and self-care of individuals.^19^ From birth to death, care is a basic human need and the primary mechanism through which health and well-being are realized. Most care globally is provided outside formal services and institutions but within families and communities.^20^ Informal care is the provision of assistance, support, or caregiving services by individuals to family members, friends, or people in need. Sources of informal care include family, friends, neighbourhoods, schools, community groups, and volunteers. For people who lack family and friends, mutually reinforcing community networks can reach and serve people through more avenues.^21^ Care can manifest in various forms, including social or emotional support, physical assistance, practical help, and kindness.

In recent decades, there has been an increasing recognition of the importance of social determinants of health, increasing efforts to integrate social welfare and healthcare services, promote community-based care, and prioritize preventative primary care and early intervention.^17^ However, these efforts primarily centre on formal (paid and regulated) care systems, and similar efforts to modernize and update the mechanisms for informal care within families and communities have not received similar attention.^20^ The availability or lack of informal care is largely invisible within formal healthcare systems. The intentional organization of informal care (self-care, family, friends, neighbours, and community) alongside formal healthcare services could address a much broader spectrum of unmet needs,^21,22^ improving both health and value for people, communities, and healthcare systems. However, limited knowledge is available regarding how to design and implement high impact, high value programs of this nature.

## Foundations for healthcare system transformation

The Health and Wellness Friendly Community Action Learning Lab (HWFC) is a voluntary research consortium dedicated to growing resources and communities that promote the physical, mental, and social health and well-being of people—see https://www.hwfc.ca/ for details. HWFC operates based on policy and practice guidance from the University of Windsor, McMaster University, the University of Toronto, and provincial organizations such as Hospice Palliative Care Ontario. HWFC researchers investigate models and exemplar programs around the world that organize social, community, and informal care alongside formal care. The following programs stand out in terms of their potential to significantly improve both population and economic outcomes.

### Programs from Canada

The Windsor-Essex Compassion Care Community (WECCC) is an ongoing pilot program offered by Community Support Services of Essex County since 2021 and formerly offered by the Hospice of Windsor-Essex between 2017 and 2020. Trained students or volunteers work one-on-one with people to self-rate their health and well-being, set goals, navigate resources, and visualize their informal and formal network of care. WECCC was originally part of a broader compassionate community initiative supported by community agencies, residences (e.g., seniors’ apartment buildings), churches, shelters, family services, provincial home care services, primary care, emergency medical services, social services, the City of Windsor, and the University of Windsor. A published program evaluation study reported that between 2017 and 2020, WECCC engaged over 2,500 program participants, 65 organizations, and 400 volunteers. Results included high satisfaction for both participants and service providers and statistically significant pre-post patient-level improvements in self-rated health, mental health, ability to do usual activities, wellness behaviour, social support, and reductions in loneliness and self-reported Emergency Department (ED) use.^23^ Evidence from a separately reported qualitative study suggested that the program acted as a safety net for people experiencing vulnerabilities, such as low income, housing insecurity, and poor social support and that it empowered people to improve their health.^24^ The principal author of this article worked extensively with the community and both sponsor agencies to review the evidence and co-design the WECCC Model; other authors were involved in supporting program evaluation efforts.

### Programs globally

The results of a community-wide social health-oriented program in Frome, United Kingdom, demonstrate dramatic potential to reduce unplanned hospitalization and healthcare costs at a population level. The initiative involved the town’s primary care practice in collaboration with a community agency funded by the National Health Service. The study of this program found a 17% reduction in unplanned hospital admissions over 5 years for the population of Frome, despite a 28.5% increase across Somerset County, where Frome is located. Additionally, Frome experienced a 21% reduction in per capita healthcare costs, accounting for 5% of their total healthcare budget. This was in contrast to a 21% increase in per capita costs across Somerset County during the same time period.^25^ A pilot study in Australia also demonstrated the benefits of reduced ED visits and the potential to reduce costs. The Compassionate Connectors Program in Australia is a community-wide compassionate community initiative delivered through the hospice sector and trained community volunteers.^26^

These and similar programs show the potential to improve outcomes by reallocating existing resources and using volunteers and community partnerships strategically. These approaches go beyond traditional services to include self-care and informal community-based actions, aiming to prevent physical, mental, and social issues rather than waiting to respond to them. By leveraging informal care networks and empowering individuals to improve self-care, programs such as WECCC,^24^ Frome,^25^ and Compassionate Connectors Australia^26^ demonstrate that it is possible to alleviate strain on healthcare services, resulting in significant cost savings and improved quality of life for individuals. Community benefits include nurturing healthy and safe social environments.

## Considerations for blueprint design and program development

Healthcare systems have a role in sustaining effective program interventions and in mainstreaming integrated informal and formal care as a routine part of population health, serious illness, and elder care delivery to achieve population impacts aligned to the Quintuple Aim.^27^ Based on lessons learned to date, generalizable features and high value practices derived from exemplar programs around the world include the following.

Integrated informal-formal care programs should include elements that work at multiple levels for high impact:

• *Individuals*: Navigation and care planning programs that include support for people to identify their unique practical, social, mental, and physical health needs and goals; support to organize a personalized network of care; informal care navigation to reduce unmet needs; and advance person-directed opportunities to address needs.
• *Groups*: Social and education programs that build a heightened sense of social connection, trust, belonging, and collaboration amongst participants and external partner agencies.
• *Agencies*: Community partnerships and coordination across sectors that reorient models of care towards wellness and improve wrap-around care for people with complex needs.
• *Healthcare system and population level*: Collective action to develop new resources and forms of support, informed by data, that shift healthcare systems towards upstream prevention, early intervention, and community networks of care.

Demonstrated processes, tools, and training programs to support implementation include:

• *Individuals*: Structured risk screening and assessment for social, mental, and physical well-being, goal setting, personalized care planning, and holistic care networks.
• *Wellness support*: Activities such as socialization, education, and informal resource linkage.
• *Coordinated services (wrap-around care)*: Partnerships with organizations, cross-organization referrals, coordination of formal and informal aspects of care, and follow-up for complex needs.
• *Community development*: Capacity building—social capital, co-designing new resources to address group needs, volunteer training programs, and collaborative data-sharing platforms.

Key features and implementation structures for scaling system-wide healthcare solutions to integrate informal and formal care include:

• Coordination by a “backbone” organization to foster the cross-sector communication, alignment, shared data, and collaboration required to achieve population-level systems change. With sufficient support and resources, agencies from any healthcare or community sector (e.g., hospice organization and community health centre) could lead and provide backbone support for local implementation efforts; provincial and national level efforts could involve independent organizations such as non-profits, academic institutions, or HWFC.
• Transparency of visualized personalized care networks, integrating physical, mental, and social resources and social participation opportunities.
• Access to navigation and connection roles (trained volunteers and paid connectors) to bridge formal and informal care provision. Incorporating students who are trained and appropriately supervised can lower costs and prepare the future workforce for a person integrated approach to care without sacrificing quality.
• Engaged communities and users that co-design additional informal and social resources to address unmet needs, informed by data.
• Robust infrastructure support for virtual delivery to reach more people at lower cost.
• Policies that strengthen enabling environments, including primary care and community leadership, health and social service coordination, supportive built, and social environments.
• Learning healthcare systems and data to grow, transform, and improve the quality and impact of efforts.

## Discussion

This article provides a blueprint for shifting towards strengthening informal care networks in concert with efforts to improve the value of healthcare. Any one or any combination of these program elements and the use of available tools can contribute further improvements to population health and quality of life. Champions and early adopters could include individuals, groups, agencies, or networks within defined geographies or sectors/populations of interest. To embed new ways of working in existing institutions, programs can be customized for key populations, including seniors, caregivers, people with mental health needs, youth, newcomers, and people from different language communities. Standardized components, policy support, and funding to grow enabling environments will benefit all communities or people.

The concepts, processes, structures, and outcomes outlined can be adapted to address any health sector or community’s needs, and goals for change. For example, one of the best ways for existing services to deliver person-centred healthcare is to base it on a person’s goals, within the context of their social network in the communities in which they reside.^20^ Implementation pathways co-designed with the person and a variety of service sectors such as home and community, primary care, long-term care, palliative care, residential services, emergency services, mental health and addictions, public health, and social welfare allow for low-cost, customized adoption, reducing the time and effort required to implement. Variations in implementation formats can be designed to address specific vulnerabilities, complex care, or social exclusion challenges and eliminate duplication. By respecting and responding to the person’s goals and priorities, social and informal care assets can be better coordinated for integrated planning and risk management. Provider experience will be enhanced through the availability of more tools to support social inclusion, and other care needs through this type of programming. Informed by data, providers, communities, and users can be mobilized to co-design and creatively develop additional informal resources to reduce unmet needs, especially for the most vulnerable. HWFC researchers are interested in further collaboration to develop and road-test new approaches, innovations and tools, influence policy, and support longer-term research in these areas.

While an initial investment is necessary, our experience with WECCC and observations of similar programs has shown that investments in informal care yield positive returns. Implementation costs are low, as individuals, assisted by volunteers as needed, become empowered to integrate informal care resources of interest to them. Access to education, training, and volunteer and community development helps to bridge formal and informal systems. The benefits gained from improved health outcomes, reduced healthcare costs, and enhanced productivity outweigh the initial investments.

## Conclusions

Evidence supports that people who carry out meaningful roles in supportive social contexts get sick less often, suffer less disability, and recover faster from life-threatening events.^28^ Both theoretical and empirical research is increasing the understanding of the important role that informal care resources can play in fostering individual well-being and improving healthcare sustainability. A well-designed, intentionally organized, and adequately resourced informal care system can yield substantial economic advantages by promoting better health, reducing healthcare costs, enhancing productivity, addressing inequalities, fostering social and economic inclusion, and better managing the increasing demands and care needs of an ageing society.

Integrating informal and formal care will expedite the fundamental shift needed to transform healthcare from systems designed around diseases and institutions towards healthcare systems designed for people. This can primarily be achieved by leveraging existing resources, structures, and systems available in most communities and by bringing together all partners—people, groups, healthcare providers, funders, policy-makers, businesses, and researchers—to work together on solutions. It is time to invest in creating the social conditions that drive real behaviour change and address a wide range of social harms. All people, especially the most vulnerable among us, can receive the support they need to live healthier, happier, more connected lives at less cost to the public and taxpayers.
